# Sequencing the organelle genomes of *Bougainvillea spectabilis* and *Mirabilis jalapa* (Nyctaginaceae)

**DOI:** 10.1186/s12863-022-01042-0

**Published:** 2022-04-13

**Authors:** Fang Yuan, Xiaozhong Lan

**Affiliations:** Tibetan Collaborative Innovation Center of Agricultural and Animal Husbandry Resources, Food Science College, TAAHC-SWU Medicinal Plant Joint R&D Center, Tibet Agriculture & Animal Husbandry University, Nyingchi, Tibet China

**Keywords:** *Mitochondrial genome*, *Chloroplast genome*, *Phylogenetics*, *Traditional medicine*, *Ornamental plants*, *DNA barcoding*

## Abstract

**Objectives:**

*Mirabilis jalapa* L. and *Bougainvillea spectabilis* are two *Mirabilis* species known for their ornamental and pharmaceutical values. The organelle genomes are highly conserved with a rapid evolution rate making them suitable for evolutionary studies. Therefore, mitochondrial and chloroplast genomes of *B. spectabilis* and *M. jalapa* were sequenced to understand their evolutionary relationship with other angiosperms.

**Data description:**

Here, we report the complete mitochondrial genomes of *B. spectabilis* and *M. jalapa* (343,746 bp and 267,334 bp, respectively) and chloroplast genomes of *B. spectabilis* (154,520 bp) *and M. jalapa* (154,532 bp) obtained from Illumina NovaSeq. The mitochondrial genomes of *B. spectabilis* and *M. jalapa* consisted of 70 and 72 genes, respectively. Likewise, the chloroplast genomes of *B. spectabilis* and *M. jalapa* contained 131 and 132 genes, respectively. The generated genomic data will be useful for molecular characterization and evolutionary studies.

## Objective

Organelle genomes such as chloroplast and mitochondrial genomes are highly conserved in plant species except for minor structural rearrangements reported in few species [1]. The conserved nature and rapid evolution rate of organelle genomes play a key role in understanding the evolutionary aspects of different species [2]. Chloroplast genomes generally have a quadripartite structure ranging from 107 to 217 kb [3]. In contrast, mitochondrial genomes are bigger in size (105 kb to 110 Mb) [4]. Compared to the nucleic genome, organelle genomes are ideal for studying phylogenetics [5–9].

The Nyctaginaceae family, known for its ornamental value and pharmaceutical properties, consists of hermaphroditic trees, shrubs, and herbs. *M. jalapa* and *B. spectabilis* originated from tropical America and have been widely adapted as ornamental plants for their vibrant colors, medicinal characteristics, and phytoremediation properties [10–14]. Antioxidative, antimicrobial, antibacterial, and antiviral effects of both species have also been reported [10, 15, 16]. Although both species have been well characterized for their bioactive components, genomic resources for molecular characterization and evolutionary analyses are rare in *M. jalapa* and *B. spectabilis*. In this study, we sequenced the chloroplast and mitochondrial genomes of *M. jalapa* and *B. spectabilis*. The generated datasets will be used to investigate the structural organization of their organelle genomes and the phylogenetic relationship with existing angiosperms.

## Data description

The leaf samples from *B. spectabilis* and *M. jalapa* were collected from Qiannan Buyi and Miao Autonomous Prefecture (N: 26° 22 ′ 75.63 ″, E:107° 62 ′ 39.08 ″), Guizhou Province, China. The samples were obtained from the wild and no permissions were necessary to collect such samples. The formal identification of the samples was conducted by Prof Xiaozhong Lan and voucher specimens were deposited at Tibet Agriculture and Animal Husbandry University (http://www.taaas.org) under the voucher numbers: ZY20-082,503 and ZY20-082,504. The total genomic DNA (gDNA) was isolated from fresh leaf samples with the CTAB method using the Plant Genomic DNA Kit (DP305, TIANGEN, China). After the fragmentation of DNA, 300 bp short insert libraries were constructed. The expected size profile was verified using gel electrophoresis. The gDNA was sequenced on the Illumina NovaSeq 6000 platform at Wuhan bio-mall Biotechnology Co., Ltd (Wuhan, China), following the standard protocols. Quality control was performed using fastqc and NGSQC, and raw data were cleaned for low-quality reads. Chloroplast and mitochondrial genomes were assembled using SPAdes v3.9.0 [17] and MITObim v1.8. The annotation was performed using CpGAVAS [18].

The obtained circular mitochondrial genomes of *B. spectabilis* and *M. jalapa* were 343,746 bp and 267,334 bp long, respectively (Data files 1 and 2). GC contents in *B. spectabilis* and *M. jalapa* mitochondrial genomes were estimated to be 37% and 34.5%, respectively. *B. spectabilis* mitochondrial genome was annotated with 70 genes. Among these, we identified 42 protein-coding genes, 25 tRNA, and three rRNA. *M. jalapa* mitochondrial genome consisted of 72 genes with 40 protein-coding genes, 28 tRNA, and three rRNA. Strong evidence of expression supported most annotated genes.

The sequenced chloroplast genomes of *B. spectabilis* and *M. jalapa* were 154,520 bp (35.9% GC content) and 154,532 bp (35.9% GC content) long, respectively (Data file 3 and 4). The quadripartite structure of *M. jalapa* chloroplast genome contained two inverted repeats regions (25,428 bp), one large-single copy (85,908 bp), and one small-single copy (17,768 bp). A total of 131 genes were identified, including 86 protein-coding genes, eight rRNA, and 37 tRNA. The chloroplast genome of *B. spectabilis* encoded 132 genes, including 87 protein-coding genes, eight rRNA genes, and 37 tRNA genes. *RPS12* gene had a trans-splicing in the two species. Similarly, in both genomes, a total of 15 genes (*trnKUUU*, *rps16*, *trnG-UCC*, *atpF*, *rpoC1*, *trnL-UAA*, *trnV-UAC*, *petB*, *petD*, *rpl16*, *rpl2*, *ndhB*, *trnI-GAU*, *trnA-UGC*, and *ndhA*) had a single intron while two genes (*clpP* and *ycf3*) had two introns.

The genomic data presented here are the first publicly available organelle genomes of *B. spectabilis* and *M. jalapa.* The datasets can be further exploited to investigate the evolutionary relationship of *B. spectabilis* and *M. jalapa* with existing Nyctaginaceae species and other angiosperms. It can also be used for the development of molecular markers and DNA barcoding applications.

### Limitations

Organelle genomes have a lower mutation rate as compared to nucleic genomes. Therefore, organelle genomes are not suitable for studying differentiation within the species (Table 1).

**Table 1:** Overview of data files/data sets

Label	Name of data file/data set	File types (file extension)	Data repository and identifier (DOI or accession number)
Data set 1	Illumina NovaSeq of *Bougainvillea spectabilis* mitochondrial genome	Fasta file	GenBank NCBI (MW167296) [19]
Data set 2	Illumina NovaSeq of *Mirabilis jalapa* mitochondrial genome	Fasta file	GenBank NCBI (MW295642) [20]
Data set 3	Illumina NovaSeq of *Bougainvillea spectabilis* chloroplast genome	Fasta file	GenBank NCBI (MW167297) [21]
Data set 4	Illumina NovaSeq of *Mirabilis jalapa* chloroplast genome	Fasta file	GenBank NCBI (MW894644) [22]

## Data Availability

The data sets are openly available in GenBank of NCBI at https://www.ncbi.nlm.nih.gov/nuccore/MW167296 (data set 1; Bio-project PRJNA682652) [19], https://www.ncbi.nlm.nih.gov/nuccore/MW295642 (data set 2; Bio-project PRJNA692028) [20], https://www.ncbi.nlm.nih.gov/nuccore/MW167297 (data set 3; Bio-project PRJNA682652) [21], and https://www.ncbi.nlm.nih.gov/nuccore/MW894644 (data set 4; Bio-project PRJNA720802) [22].

## Abbreviations

GC: Guanine-Cytosine
rRNA: Ribosomal ribonucleic acid
tRNA: Transfer ribonucleic acid
CTAB: Cetyltrimethylammonium bromide
DNA: Deoxyribonucleic acid

## References

[1] Xu X, Wang D (2021). Comparative chloroplast genomics of corydalis species (papaveraceae): evolutionary perspectives on their unusual large scale rearrangements. Front Plant Sci.

[2] Negruk V (2013). Mitochondrial genome sequence of the legume Vicia faba. Front Plant Sci.

[3] Park S, Ruhlman TA, Sabir JS, Mutwakil MH, Baeshen MN, Sabir MJ, Baeshen NA, Jansen RK (2014). Complete sequences of organelle genomes from the medicinal plant rhazya stricta (apocynaceae) and contrasting patterns of mitochondrial genome evolution across asterids. BMC Genomics.

[4] Wynn EL, Christensen AC. Repeats of unusual size in plant mitochondrial genomes: identification, incidence and evolution. G3. 2019;9(2):549–59. 10.1534/g3.118.200948 PMC6385970 30563833

[5] Tillich M, Lehwark P, Pellizzer T, Ulbricht-Jones ES, Fischer A, Bock R, Greiner S (2017). GeSeq–versatile and accurate annotation of organelle genomes. Nucleic Acids Res.

[6] Rawal HC, Kumar PM, Bera B, Singh NK, Mondal TK (2020). Decoding and analysis of organelle genomes of Indian tea (camellia assamica) for phylogenetic confirmation. Genomics.

[7] Lee JM, Song HJ, Park SI, Lee YM, Jeong SY, Cho TO, Kim JH, Choi H-G, Choi CG, Nelson WA (2018). Mitochondrial and plastid genomes from coralline red algae provide insights into the incongruent evolutionary histories of organelles. Genome Biol Evol.

[8] Wang S, Yang C, Zhao X, Chen S, Qu G-Z (2018). Complete chloroplast genome sequence of Betula platyphylla: gene organization, RNA editing, and comparative and phylogenetic analyses. BMC Genomics.

[9] Nazir MF, He S, Ahmed H, Sarfraz Z, Jia Y, Li H, Sun G, Iqbal MS, Pan Z, Du X (2021). Genomic insight into the divergence and adaptive potential of a forgotten landrace G hirsutum L purpurascens. J Genet Genomics.

[10] Gogoi J, Nakhuru KS, Policegoudra RS, Chattopadhyay P, Rai AK, Veer V (2016). Isolation and characterization of bioactive components from Mirabilis jalapa L radix. J Tradit Complement Med.

[11] Polturak G, Heinig U, Grossman N, Battat M, Leshkowitz D, Malitsky S, Rogachev I, Aharoni A (2018). Transcriptome and metabolic profiling provides insights into betalain biosynthesis and evolution in Mirabilis jalapa. Mol Plant.

[12] Abdel-Salam OM, Youness ER, Ahmed NA, El-Toumy SA, Souleman AM, Shaffie N, Abouelfadl DM (2017). Bougainvillea spectabilis flowers extract protects against the rotenone-induced toxicity. Asian Pac J Trop Med.

[13] Do LT, Aree T, Siripong P, Pham TN, Nguyen PK, Tip-Pyang S (2016). Bougainvinones A-H, peltogynoids from the stem bark of purple Bougainvillea spectabilis and their cytotoxic activity. J Nat Prod.

[14] Li Q, Wang H, Wang H, Wang Z, Li Y, Ran J, Zhang C (2020). Re-investigation of cadmium accumulation in Mirabilis jalapa L: evidences from field and laboratory. Environ Science Pollut Res.

[15] Sharma HK, Chhangte L, Dolui AK (2001). Traditional medicinal plants in Mizoram. India Fitoterapia.

[16] Chauhan P, Mahajan S, Kulshrestha A, Shrivastava S, Sharma B, Goswamy H, Prasad G (2016). Bougainvillea spectabilis exhibits antihyperglycemic and antioxidant activities in experimental diabetes. Journal of evidence-based complementary & alternative medicine.

[17] Nurk S, et al. Assembling Genomes and Mini-metagenomes from Highly Chimeric Reads. In: Deng M, Jiang R, Sun F, Zhang X. (eds). Research in Computational Molecular Biology. RECOMB 2013. Lecture Notes in Computer Science. vol. 7821. Berlin: Springer; 2013. 10.1007/978-3-642-37195-0_13.

[18] Hahn C, Bachmann L, Chevreux B (2013). Reconstructing mitochondrial genomes directly from genomic next-generation sequencing reads—a baiting and iterative mapping approach. Nucleic Acids Res.

[19] Yuan F, Lan X. Illumina NovaSeq of *Bougainvillea spectabilis* mitochondrial genome. GenBank of NCBI. 2021. https://www.ncbi.nlm.nih.gov/nuccore/MW167296 (PRJNA682652).

[20] Yuan F, Lan X. Illumina NovaSeq of *Mirabilis jalapa* mitochondrial genome. GenBank of NCBI. 2021. https://www.ncbi.nlm.nih.gov/nuccore/MW295642 (PRJNA692028).

[21] Yuan F, Lan X. Illumina NovaSeq of *Bougainvillea spectabilis* chloroplast genome. GenBank of NCBI. 2021. https://www.ncbi.nlm.nih.gov/nuccore/MW167297 (PRJNA682652).

[22] Yuan F, Lan X. Illumina NovaSeq of *Mirabilis jalapa* chloroplast genome. GenBank of NCBI. 2021. https://www.ncbi.nlm.nih.gov/nuccore/MW894644 (PRJNA720802).

